# CDC Guidance for Community Assessment and Investigation of Suspected Suicide Clusters, United States, 2024

**DOI:** 10.15585/mmwr.su7302a2

**Published:** 2024-02-29

**Authors:** Eva Trinh, Asha Z. Ivey-Stephenson, Michael F. Ballesteros, Nimi Idaikkadar, Jing Wang, Deborah M. Stone

**Affiliations:** 1 Division of Injury Prevention, National Center for Injury Prevention and Control, CDC, Atlanta, Georgia

## Abstract

*This report is the second of three reports in the* *MMWR* *supplement updating CDC’s guidance for investigating and responding to suicide clusters. The first report,* Background and Rationale — CDC Guidance for Assessing, Investigating, and Responding to Suicide Clusters, United States, 2024*, describes an overview of suicide clusters, methods used to develop the supplement guidance, and intended use of the supplement reports. The final report,* CDC Guidance for Community Response to Suicide Clusters, United States, 2024*, describes how local public health and community leaders can develop a response plan for suicide clusters. This report provides updated guidance for the approach to assessing and investigating suspected suicide clusters. Specifically, this approach will guide lead agencies in determining whether a confirmed suicide cluster exists, what concerns are in the community, and what the specific characteristics are of the suspected or confirmed suicide cluster. The guidance in this report is intended to support and assist lead agencies and their community prepare for, assess, and investigate suicide clusters. The steps provided in this report can be adapted to the local context, culture, capacity, circumstances, and needs for each suspected suicide cluster.*

## Introduction

Suicide is among the 10 leading causes of death among persons aged 10–64 years, and the age-adjusted rates of suicide for overall population of all ages increased approximately 36%, from 10.4 suicides per 100,000 population in 2000 to 14.1 in 2021 (*1*,*2*). Suicide deaths are just one part of assessing the public health problem of suicide and its contributing factors (*3*,*4*). Many more persons think about, plan, and attempt suicide (*3*–*6*). Monitoring these suicide-related events is a key component of prevention and might reveal when an unusual pattern or a cluster of suicides or suicide attempts have occurred.

Suicide clusters are a group of suicides or suicide attempts that occur closer together in time, space, or both than would normally be expected in a community (*7*,*8*). The two most common types of clusters reported are point clusters (i.e., spatial-temporal clusters) and mass clusters (i.e., temporal clusters) (*9*). Point clusters occur in a defined geographic location (e.g., school, institution, county, or tribe). Clusters also might be geographically dispersed over long distances (e.g., after a celebrity suicide), which is known as a mass cluster (*10*–*15*).

**FIGURE 1:**
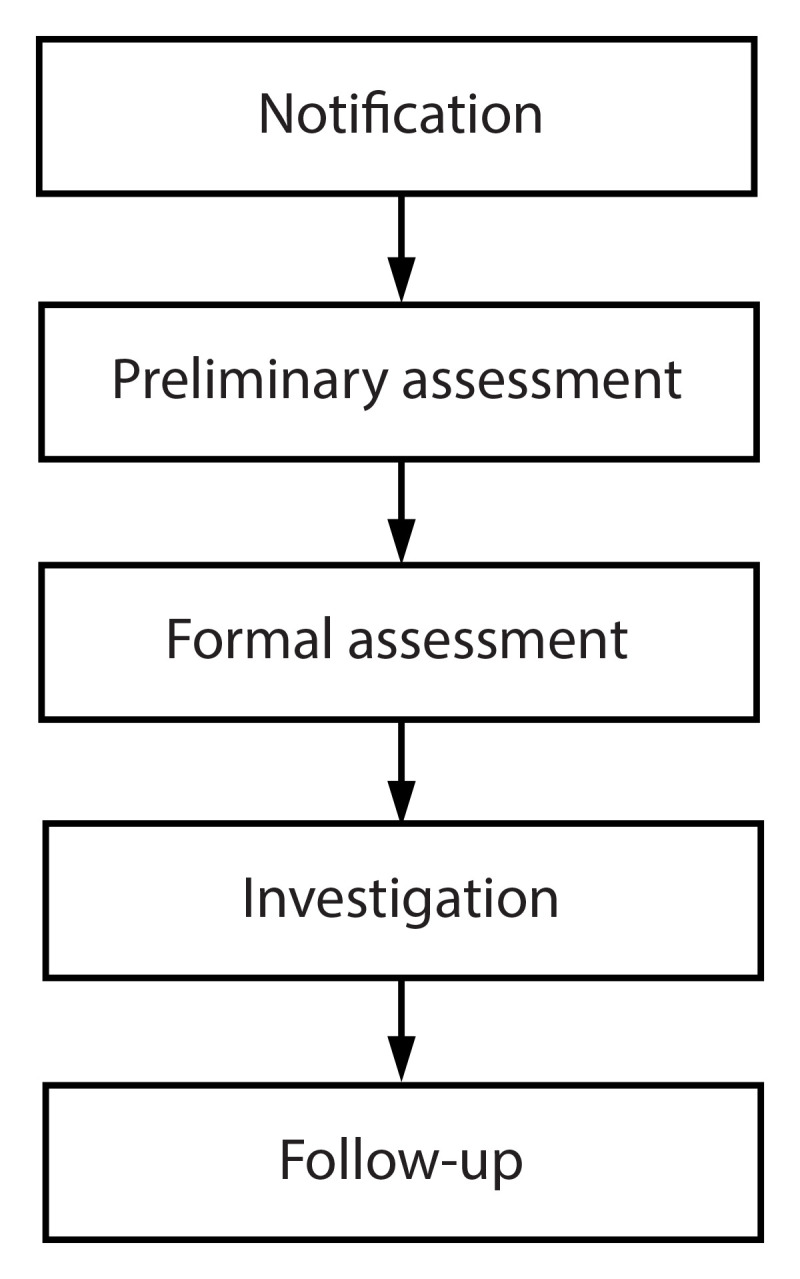
Steps to assess suspected suicide clusters, CDC guidance, 2024* * In certain instances, initiation of a community response simultaneously during the other steps might be warranted.

Notification of a potential suicide cluster, assessing and investigating a suspected suicide cluster, and responding to a cluster by taking community-specific actions might stop a suicide cluster from continuing and might prevent further suicides and any other effects related to the suspected suicide cluster (*9*,*16*,*17*). Although suicide clusters are rare, their presence or suspected presence can create a high degree of anxiety and fear of future deaths or injuries in communities (*18*). Steps can be taken within communities to help assuage this anxiety and assist with preparing for an assessment and investigation of a suspected cluster as well as a community response, if needed (Figure 1). Further, these efforts do not stop with a defined cluster or community response because follow-up assessments, investigations, and updated responses might continue as needed.

## Receiving Notification About a Potential Suicide Cluster

Notification of a suspected suicide cluster comes to an agency’s attention through a various sources. External sources can include local community partners, schools, hospitals, medical examiner or coroner’s offices, other institutions or departments, news media, suicide prevention practitioners, state suicide prevention offices, and concerned citizens. Sources that are internally a part of the agency might include, but are not limited to, suicide surveillance groups and public health or other officials’ tracking and monitoring suicide, suicide attempts, and other suicide-related outcomes (*19*–*21*). When a notification is received by the lead agency (defined as the agency that receives or acts upon the notification of a suspected cluster), the agency might use the guidance to determine action steps.

## Approach to Assess and Investigate a Suspected Suicide Cluster

**FIGURE 2:**
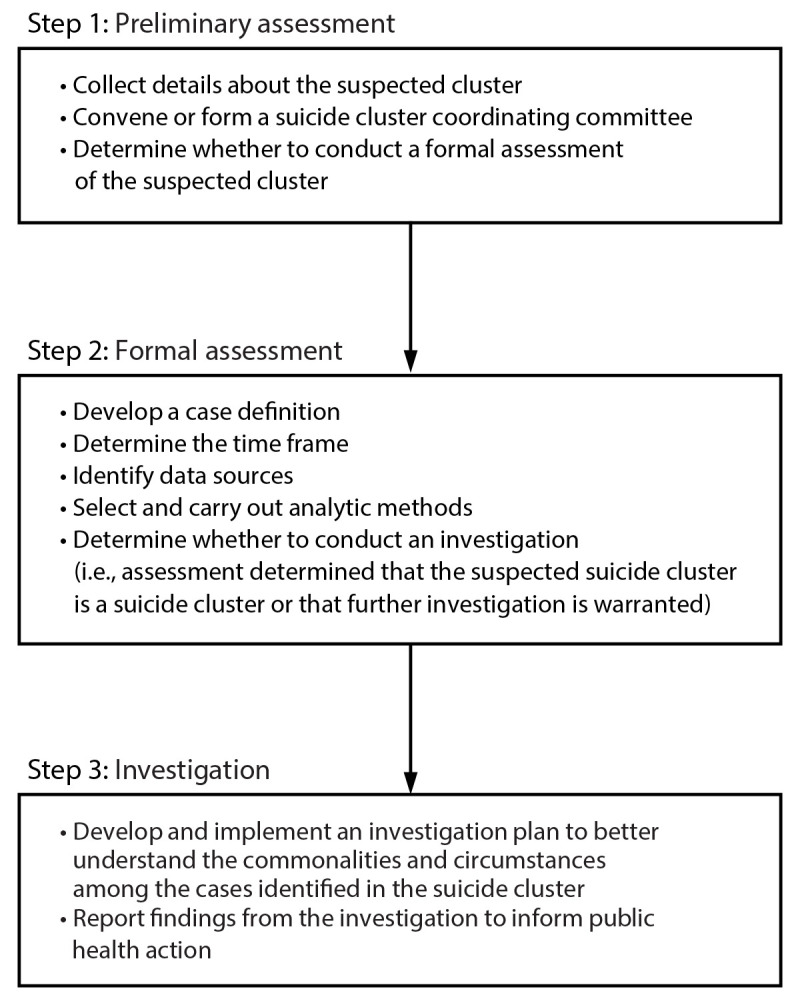
Steps to assess and investigate a suspected suicide cluster, CDC guidance, 2024

A lead agency can use a three-step approach when notified of a suspected suicide cluster (Figure 2). The first step is to conduct a preliminary assessment of the information obtained to determine whether a formal assessment is warranted. The second step is to conduct a formal assessment of the suspected cluster to determine whether it meets the definition of a cluster. The third step is to conduct an investigation to identify commonalities or similarities in cases that can guide a community response. Each lead agency might adapt the steps to fit their community’s capacity, circumstances, and needs. This report uses the terms assessment and investigation to signify different actions. Assessment is used to describe analyses that might determine if a suspected suicide cluster is a suicide cluster. Investigation is used to describe further analyses to better understand the commonalities and circumstances among the cases identified in the suicide cluster. In certain instances, initiating a community response simultaneously during any of the steps might be warranted. Information from each of the steps can be incorporated into plans for the provision or augmentation of specific services throughout this process.

### Step 1: Preliminary Assessment of Suspected Suicide Cluster

In Step 1, the lead agency reviews information about a suspected cluster and determines, in collaboration with a suicide cluster coordinating committee (committee) (Step 1B), whether a formal assessment is necessary.

#### 1A: Collect Details About the Suspected Cluster

When the notification is received, the agency collects the contact’s name, email address, phone number, and organization or other affiliation. The agency can share the information with other investigators and collaborators that are a part of the committee (Step 1B), the community, or media while upholding and respecting anonymity and confidentiality to the greatest extent, in light of how much of the decedents’ information has been communicated. The lead agency might designate one staff member as the point of contact. The responsibility of this person, sometimes referred to as the suicide cluster liaison (liaison) in the initial phase, is to collect and review the information about the suspected cluster. For this role to pre-exist is preferable but, if not, this role can be designated when the notification of the suspected cluster occurs.

The information collected about the suspected suicide cluster can include:

- Number of cases
- Types of cases (e.g., suicides and suicide attempts)
- Period when the cases occurred (e.g., past 6 months, date of the last known suicide, and whether the suspected cluster is ongoing)
- Source of information obtained for possible use in verifying the cases
- Geographic location, which also might include population density and demographic profile of the area
- Physical location of cases (e.g., wooded area, school campus, and railroad tracks)
- Mechanism of suicide among cases
- Relation of cases to each other, if known, and whether any exists
- Demographic characteristics of the cases (e.g., decedent’s age, sex, race and ethnicity, sexual orientation, and gender identity)
- Risk factors or any precipitating circumstances or events (e.g., mental health condition and exposure to other suicide attempts or deaths by suicide), if known

#### 1B: Convene or Form a Committee

The liaison convenes a pre-existing committee or forms a new committee. Members of the committee might include a combination of suicide subject matter experts, epidemiologists, data analysts, representatives of other state or local agencies, tribal partners, community partners, local suicide prevention coalition members, persons with lived experience, advocacy groups, funders, mental health organizations, health care providers, community leaders, and others who can contribute critical information related to the reported suspected cluster. The lead agency also can contact CDC to support the assessment (https://wwwn.cdc.gov/dcs/ContactUs/Form). CDC can send resource materials, provide technical assistance, or be more extensively involved through an Epidemiologic Assistance investigation (https://www.cdc.gov/eis/request-services/epiaids.html). The role of the committee, working alongside the liaison, is to

- review the available information on the suspected cluster and determine whether to proceed to a formal assessment (Step 2);
- implement the assessment, if necessary;
- contribute to a more formal investigation (Step 3), if necessary;
- provide input about the needs and concerns of the community; and
- be involved in the day-to-day activities, as requested.

When engaging community partners and organizations, following communication best practices to avoid any miscommunication or misinterpretation about the suspected cluster is important. Taking caution in media messaging that can inadvertently increase the risk for suicide contagion (i.e., occurrence of additional suicide attempts by those who have been exposed to suicide or suicidal behavior), which is a concern when dealing with suspected suicide clusters (*10*,*22*,*23*).

At each step in the process, the membership of the committee might change depending on the need for more specialized expertise or based on completion of tasks. The liaison, along with the existing committee, might determine necessary changes in group membership as the process unfolds. New members of the committee might be internal or external to the lead agency and might include epidemiologists, communicators, staff members from local crisis centers supporting the national 988 Suicide & Crisis Lifeline (call or text 988 or go to https://988lifeline.org), statisticians, mental health experts, other suicide prevention experts, and others with specific expertise as needed. As new members are added, they can be provided with information about the expectations and goals of the committee along with a summary of all activities and information collected.

#### 1C: Determine Whether to Conduct a Formal Assessment of the Suspected Cluster

The committee, led by the liaison in the lead agency, can review the details of the suspected cluster and engage in initial discussions to determine whether to conduct a formal assessment (Step 2). Guiding questions might include:

- Do the number of cases and information received present a concern for a cluster rather than a general increase in cases?
- Is there evidence to suggest the cases are connected to each other in time and space (e.g., geographically, physically, and virtually) by specific risk factors or by way of common demographic characteristics?
- Has information been shared in the community?

If the committee determines that sufficient justification or evidence exists, then they can proceed to Step 2 to formally assess the suspected cluster. The liaison also might notify the source of information that a formal assessment will be conducted.

If the committee determines evidence is insufficient, the liaison prepares a summary report to the source that provides the initial notification and can share the report with members of the committee who participated in the deliberations. The report will clearly and accurately explain the reason why the agency is not pursuing a formal assessment or investigation based on the information provided about the cases, including any procedures that were taken, any results from the initial review of available information, any suicide prevention and intervention materials that are appropriate to share, and any referrals to other appropriate agencies that might share the reported concern (e.g., poisoning or overdose prevention programs).

### Step 2. Formal Assessment of the Suspected Suicide Cluster

In Step 2, the committee further assesses and determines whether the suspected suicide cluster warrants further investigation (Step 3). The liaison can coordinate meetings and outline steps in the assessment process. For example, the liaison, in conjunction with the committee, can develop an assessment plan that includes developing a case definition, determining a time frame for the analysis, identifying available and accessible data sources, determining the method of data collection, developing a data analysis plan, and assessing the available data and information, including selecting the appropriate statistical tests to determine if the cases in question can be classified as a true cluster. During this step, the committee can determine whether to invite additional members (e.g., epidemiologists, data analysts, behavioral scientists, and survey developers).

#### 2A: Develop a Case Definition

A definition of what is considered a case is needed to effectively assess whether a cluster has occurred. This definition should, if possible, include the specific type of event (e.g., suicide or suicide attempt), a specific time frame, geographic boundaries (e.g., county, town, school district, and region of the state), specific population characteristics (e.g., sex, age, specific school or school district, and occupation), and means of suicide, as appropriate.

#### 2B: Determine the Time Frame

Determining the time frame of the suspected cluster is needed for the assessment. For the committee to determine whether the suicide cluster is ongoing or restricted to the reported cluster period to guide response action is important. Assessing an increase in suicide should be conducted for the suspected cluster period and for the period thereafter up to the most recent time if data are available. Determining a baseline period for the assessment helps ensure accurate and valid comparison of the time frame of suspected cluster. For example, the committee might set a baseline to compare any changes in the suspected cluster period reported (e.g., comparing months in the previous year[s] with months in the current year). A baseline also can be the same weeks or months in previous years to account for seasonal suicide trends (*24*).

#### 2C: Identify Data Sources

The decision on which data are appropriate for this stage is determined by the suspected type of suicide cluster (e.g., deaths versus attempts or point cluster versus mass cluster) and other characteristics of the suspected cluster (e.g., specific population subgroup or common method used) (Box 1). When determining which data sources to use, the following questions might be considered:

BOX 1 **Data sources for assessment and investigation of suspected suicide clusters, CDC guidance, 2024**

**Suicide deaths and precipitating circumstances:**

- Death certificate data
- Child fatality reviews data
- State violent death reporting system
- Coroner or medical examiner reports
- Law enforcement reports
- Toxicology reports
- State or local health information exchange or data warehouse

**Suicidal ideation, self-harm, or suicide attempt:**

- Emergency department or hospitalization data (e.g., Electronic Surveillance System for the Early Notification of Community-Based Epidemics)
- Syndromic surveillance data
- Emergency medical services data
- Medicare and Medicaid data
- State and local suicide prevention lifeline data (e.g., 988)
- Poison control center data
- Social media and other online data (e.g., social media surveillance data and online safety monitoring data)

**Risk or protective factors for suicide:**

- Youth Risk Behavior Surveillance System (at local level, if available)
- Behavioral Risk Factor Surveillance System (at local level, if available)
- Child protective services records
- Court data related to domestic violence, legal involvement
- Local mental health care data
- Prescription drug monitoring programs or other state and local drug use data
- Local and national newspapers

**Population size that can be used as denominator to calculate rates:**

- CDC’s Wide-ranging Online Data for Epidemiologic Research (WONDER)

**Suicidal behaviors and risk factors at state and national level for references:**

- CDC’s WONDER
- CDC’s Web-based Injury Statistics Query and Reporting System (WISQARS)
- National Survey on Drug Use and Health
- Federal Reserve economic data
- General social survey
- Census housing data

- Are data available on the outcome of interest?
- Are data available at the appropriate geographic level to draw meaningful insights?
- How recent are the data and do they cover the cluster period?
- Do the data include the baseline period?
- What is needed to access the data (e.g., mechanisms of data transfer, permissions)?
- Can data from different data sources be linked to provide additional information?
- Can emerging technologies be leveraged to gain more insight or knowledge into the cluster (e.g., social media and other online data)?

#### 2D: Select Statistical and Analytical Methods

The persons on the committee who are tasked with data analysis might conduct relatively simple analyses (e.g., descriptive analyses) or more complex analyses. Analytic methods can vary depending on the characteristics of the suspected cluster and key questions to be addressed. Assessment of the suspected cluster can include comparing trends over time, using inferential or health statistics, and conducting analyses that incorporate both quantitative and qualitative methods. Because a suicide cluster generally refers to a short-term increase in suicides or attempts, monthly or even weekly counts might be the analytic unit ascribed to precisely define the period for assessment. A visual trend of the number of cases over a specified period can be the first step in determining whether an increase or emerging pattern exists. Analyzing behavioral trends over time also can be useful in determining whether simultaneous or similar trends are occurring in risk behaviors associated with suicide (e.g., self-harm and suicidal ideation).

The ability to produce statistically reliable estimates and detect a significant increase in suicide outcomes might be hampered when assessing small numbers of cases. This limitation is not uncommon in analyses of clusters. To overcome certain of these challenges, the committee or persons conducting the analyses can consider analytic methods that account for small numbers and the spatial-temporal nature of the data. Statistical methods that can quantitatively detect suicide clusters are described in a review (*25*). Common methods might include, but are not limited to:

- Space-time permutation scan statistic: The space-time scan statistic uses a space-time permutation probability model; a cylinder scanning window focuses on geographic areas and a height mirroring time (*26*,*27*). The significant clusters will represent overlapping cylinder shapes of geography and time. The recommended platform used to perform a space-time permutation scan statistic is SaTScan, a software developed by Martin Kulldorff and Information Management Services Inc. SaTScan is both a spatial and space-time scan statistic platform.
- Bayesian spatial temporal analysis: Bayesian hierarchical models provide the ability to explore and analyze spatial-temporal health data. Recent advances in technology and computing make this method more accessible for analysts to perform. This method relies on a probability model that uses observed and population distribution to calculate the posterior distribution for significant clusters (*28*).
- Poisson probability density function: This approach uses a Poisson distribution to estimate the probability of observing the number of cases reported in the suspected suicide cluster based on the rate in the reference period (*25*,*29*). The average count of cases per analytic unit (e.g., per month) during the reference period is obtained. The p value is then used to provide statistical support for the occurrence of the suicide cluster.
- Anderson-Darling test: This approach tests whether a potential suicide cluster differs from that which would be expected under a homogeneous Poisson distribution (*25*,*30*). The Anderson-Darling test is especially useful for identifying clustering in small samples.

The committee will review the findings and limitations of this initial assessment to determine whether further investigation should be conducted.

#### 2E: Determine Whether to Conduct Further Investigation

If a cluster is confirmed, the committee can proceed to an investigation. If analytic testing does not demonstrate a greater than expected increase, an investigation might still be warranted, especially if the community perceives that a suicide cluster exists or perceives heightened anxiety in the community that could lead to potential contagion. If the committee determines the cases represent an isolated or sporadic marginal increase (e.g., not a statistically significant increase), they might decide to continue monitoring the suspected cluster rather than conduct an investigation.

If the committee agrees that there is no concern for a suicide cluster, then the liaison should prepare a summary report to the sources that provided the initial notification. The committee should assist in developing the summary report of the findings and include a justification for why an investigation is not recommended and provide information about any other steps that will be taken (e.g., continued monitoring, communication, or suicide prevention education within the community).

### Step 3. Investigation of the Suicide Cluster

The purpose of this step is to investigate potential commonalities or precipitating circumstances among the cases. This information can be used in the community response plan (*31*) and can contribute to epidemiologic and public health knowledge. During this step, the committee can invite additional members including, but not limited to, epidemiologists, behavioral scientists, and community representatives.

#### 3A: Develop and Implement an Investigation Plan

In this step, the committee will develop and implement an investigation plan for analyzing risk and protective factors among cases and identifying any patterns in precipitating factors or events in the suicide cluster. Under the leadership of the liaison, the committee will determine an appropriate and feasible study design for the investigation that addresses objectives and hypotheses formulated by the committee. Considerations for the depth and breadth of the investigation might include availability of resources and staff member time, size of the cluster, geographic spread, period being examined, availability of data, and limitations of the data.

The investigation plan might include reviewing the literature on suicide clusters, specifically paying attention to known risk factors, circumstances, and contributors of suicide (*32*,*33*). This review and discussions among the committee can inform establishing the investigation’s objectives and specific hypotheses. The investigation plan also might include assessing the availability of existing data (Box 1) that can be examined at the individual, relationship, community, or societal level (e.g., demographic characteristics, risk factors [e.g., school, job, or financial problems], exposure to a suicide in the community, and engagement in a suicide online forum) (Box 2). Other relevant data can be abstracted from case notes (e.g., medical examiner, coroner, law enforcement, or first-responder reports), toxicology reports, and hospital triage notes. In addition, media scans, social media, and other online data can provide additional information on the local situation. To better understand the suicide cluster, the investigation also might include factors specific to the community (e.g., the community’s values and culture, attitudes toward suicide and help-seeking, and history of suicide prevention).

BOX 2 **Common suicide cluster variables of interest, CDC guidance, 2024**

**Demographics and descriptive characteristics:**

- Sex
- Age
- Race
- Ethnicity
- Location
- Rurality or urbanicity
- Gender identity
- Sexual orientation
- Job industry or occupation
- Veteran status
- Disability status
- Experience of homelessness
- First- or second-generation immigrant
- Mechanism of suicide

**Individual circumstances, events, or risk factors:**

- Previous suicide attempt
- Mental health conditions
- Physical health problems
- Serious illness
- Chronic pain
- Criminal or legal problems
- Job or financial problems or loss
- Impulsive or aggressive tendencies
- Problematic substance or alcohol use
- Current or previous history of adverse childhood experiences
- Recent traumatic experience
- Sense of hopelessness
- Violence victimization or perpetration
- Eviction or loss of home

**Relationship circumstances, events, or risk factors:**

- Bullying (including cyberbullying)
- Family or loved one’s history of suicide or death of a family member or friend
- Recent argument or conflict
- Relationship breakup
- Other relationship loss
- High-conflict or violent relationships
- Social isolation

**Community circumstances, events, or risk factors:**

- Lack of access to health care
- Suicide cluster in the community
- Stress of acculturation
- Community violence
- Historical trauma
- Discrimination or racism

**Societal circumstances, events, or risk factors:**

- Stigma associated with help-seeking and mental illness
- Easy access to lethal means of suicide among persons at risk
- Unsafe media portrayals of suicide

**Outcomes:**

- Suicidal ideation
- Suicide planning
- Suicide attempt
- Self-harm, including nonsuicidal self-harm
- Death by suicide

If feasible, qualitative data can be obtained through in-depth interviews with or focus groups of persons directly connected to the cases, persons with lived experience, or members of key community sectors and other interested groups (e.g., hospital and emergency response staff members, school staff members, and community groups). To obtain further understanding and insight into the cluster, interviewees might be asked whether they are aware of an increase in suicide attempts or deaths, what the suspected contributors of suicide for this cluster might be, whether there have been any observed patterns (e.g., exposure to a suicide risk factor or relationship among the decedents), what concerns the community currently has, what current programs and initiatives there are that address suicide, and how well those are working (*34*).

Determining a comparison group might be helpful for examining similarities and differences in characteristics, circumstances, and contributing factors to guide prevention. For example, suicides during the investigation period might be compared with those during the reference period for this purpose.

Developing and implementing an analysis plan might include descriptive analyses to compare the distributions of epidemiologic characteristics between recent cases with cases from previous years. Further, clustering analyses at the individual level might identify common profiles among the investigated cases that analyses at aggregated level might miss. This approach might include a hierarchical clustering analysis to develop a heatmap on profiles of characteristics or factors among the investigated suicide cases (e.g., by using the R pheatmap package developed by The R Foundation to identify distinguished patterns of clustering).

#### 3B: Report Findings to Inform Public Health Action

At the end of Step 3, the liaison and committee develop a final report describing procedures, findings, and recommendations that reflect the community’s culture and environment and decisions made each step of the way. These findings can inform a community response plan (*31*) to prevent further suicides. The liaison also might share a summary report with the information source that provided the initial notification, as well as with the public and media, as appropriate, ensuring the guidelines and best practices for safe reporting on suicide are followed.

## Conclusion

This CDC guidance is meant to support and assist communities in the assessment and investigation of suspected suicide clusters that can ultimately guide public health action (e.g., community response) to prevent suicide. Investigating a suspected cluster requires substantial time and resources for the lead agency and community. Ideally, the committee and liaison are prepared to receive concerns, engage key partners, and assess and investigate suspected clusters. This CDC guidance provides direction for a carefully planned and implemented process. Having an established approach can help an agency and community prepare the infrastructure and resources to readily act when suicide clusters are suspected or confirmed. Although suicide clusters comprise a small proportion of suicides, a suicide cluster or the perception of a suicide cluster can greatly affect communities (*18*).

Assessment and investigation of a suspected suicide cluster will vary by community based on community values and culture, attitudes toward suicide and help-seeking, history of suicide prevention efforts, and other factors. In addition, lead agencies will differ in their resources and capacity to address challenges (e.g., small sample sizes, data completeness and availability, the possibility of clusters that lack physical spatial boundaries, and an urgency to respond to a community’s perception of a suspected suicide cluster). Over time, new data sources and information will add to the analysis and insight toward cluster investigation. These community strengths and limitations can help tailor the assessment and investigation process.

Communication is a critical component throughout the assessment and investigation process. Ongoing effective communication is necessary from reliable spokespersons, both internally within the committee and externally to decision-makers and the public whether the overall investigation ends with the first or last step of this guidance. Following best practices for safe messaging and reporting on suicide makes the difference in controlling or exacerbating an already stressful situation.

Finally, the work of the liaison and committee in this phase can help guide the community response. The community response stage is when public health efforts can be implemented to save lives.
